# Regenerative Calcium Currents in Renal Primary Cilia

**DOI:** 10.3389/fphys.2022.894518

**Published:** 2022-05-10

**Authors:** Steven J. Kleene

**Affiliations:** Department of Pharmacology and Systems Physiology, University of Cincinnati, Cincinnati, OH, United States

**Keywords:** primary cilium, polycystic kidney disease, polycystin-2, PC2, TRPV4, calcium signaling

## Abstract

Polycystic kidney disease (PKD) is a leading cause of end-stage renal disease. PKD arises from mutations in proteins, one a Ca^2+^-conducting channel, expressed in the primary cilia of renal epithelial cells. A common hypothesis is that Ca^2+^ entering through ciliary ion channels may reduce cystogenesis. The cilia have at least two Ca^2+^-conducting channels: polycystin-2 (PC2) and TRPV4 (transient receptor potential (TRP) cation channel, subfamily V, member 4), but how substantially they can increase intraciliary Ca^2+^ is unknown. By recording channel activities in isolated cilia, conditions are identified under which the channels can increase free Ca^2+^ within the cilium by at least 500-fold through regenerative (positive-feedback) signaling. Ca^2+^ that has entered through a channel can activate the channel internally, which increases the Ca^2+^ influx, and so on. Regenerative signaling is favored when the concentration of the Ca^2+^ buffer is reduced or when a slower buffer is used. Under such conditions, the Ca^2+^ that enters the cilium through a single PC2 channel is sufficient to almost fully activate that same channel. Regenerative signaling is not detectable with reduced external Ca^2+^. Reduced buffering also allows regenerative signaling through TRPV4 channels, but not through TRPM4 (TRP subfamily M, member 4) channels, which are activated by Ca^2+^ but do not conduct it. On a larger scale, Ca^2+^ that enters through TRPV4 channels can cause secondary activation of PC2 channels. I discuss the likelihood of regenerative ciliary Ca^2+^ signaling *in vivo*, a possible mechanism for its activation, and how it might relate to cystogenesis.

## Introduction

Polycystic kidney disease (PKD), a hereditary disorder that often causes end-stage renal disease, is a ciliopathy. It results from mutations in proteins that are expressed in part in the cilia of renal epithelial cells (Pazour et al., 2002; Yoder et al., 2002; Ward et al., 2003; Wang et al., 2004; Zhang et al., 2004). Cystic cells are characterized by excessive secretion and proliferation, and the cystic phenotype can be greatly exacerbated by the presence of the cilium (Ma et al., 2013). Evidence indicates that control of cytoplasmic Ca^2+^ is defective in the disease. In PKD, renal epithelial cells often have reduced cytoplasmic Ca^2+^ (Yamaguchi et al., 2006; Zaika et al., 2013; Jin et al., 2014b; Tomilin et al., 2018; but see also; Cabrita et al., 2021), and restoring this Ca^2+^ can reduce the cystic phenotype (Yamaguchi et al., 2006; Gradilone et al., 2010; Zaika et al., 2013). Thus it is has been suggested that, in healthy cells, Ca^2+^ entering through ciliary ion channels helps to maintain a beneficial level of internal Ca^2+^ (Jin et al., 2014b; Chebib et al., 2015; Wang et al., 2018).

Two channels have been identified that could conduct Ca^2+^ into the renal primary cilium. The first of these is polycystin-2 (PC2, also called PKD2, TRPP2, and TRPP1). PC2 is a member of the TRPP subfamily of transient receptor potential (TRP) cation-conducting channels (Wu et al., 2010). Mutations in PC2 account for ∼15% of cases of the autosomal dominant variant of polycystic kidney disease (ADPKD) (Cornec-Le Gall et al., 2018). PC2 is expressed in both the endoplasmic reticulum (Koulen et al., 2002) and the primary cilium (Pazour et al., 2002; Yoder et al., 2002; Kleene and Kleene 2017; Liu et al., 2018). A second Ca^2+^-conducting channel functions in the primary cilium: TRPV4 (Köttgen et al., 2008; Du et al., 2012; Zhang et al., 2013; Lee et al., 2015; Kohli et al., 2017; Siroky et al., 2017), a member of the vanilloid subfamily of TRP channels. It is not clear how effectively the native PC2 and TRPV4 channels support a ciliary Ca^2+^ influx. In the resting state, ciliary PC2 allows only a very small inward Ca^2+^ current (Kleene and Kleene 2021), limited by a small single-channel conductance to Ca^2+^ (Liu et al., 2018) and a very low open probability near the resting potential (Kleene and Kleene 2017; Liu et al., 2018; Ha et al., 2020). In the cilium, TRPV4 activity has only been demonstrated following activation with an agonist (Siroky et al., 2017).

Both PC2 (Kleene and Kleene 2017; Liu et al., 2018; Kleene and Kleene 2021) and TRPV4 (Strotmann et al., 2003) conduct Ca^2+^ and are also activated by internal Ca^2+^ (Strotmann et al., 2003; Liu et al., 2018; Kleene and Kleene, 2017; Kleene and Kleene, 2021). One can speculate that a small initial activation of either channel could lead to a large Ca^2+^ signal through a regenerative (positive-feedback) mechanism. A small Ca^2+^ influx would increase the Ca^2+^ concentration at the internal face of the channel. That would further activate the channel, increasing the Ca^2+^ influx, and so on. In this report, I identify laboratory conditions that allow powerful amplification of ciliary Ca^2+^ signals through positive feedback. With reduced Ca^2+^ buffering, Ca^2+^ influx through a single PC2 channel is sufficient to drive that same channel to an almost fully open state near a typical resting membrane potential. On a larger scale, activation of TRPV4 channels can create sufficient intraciliary Ca^2+^ to secondarily activate PC2 channels. I discuss the likelihood of regenerative Ca^2+^ signaling *in vivo*. I also propose a means for its activation and relate that to a possible mechanism of cystogenesis in PKD.

## Materials and Methods

### Electrical Recording

All electrical recordings were made from whole primary cilia of mIMCD-3 cells as described previously (Kleene and Kleene 2012). In short, mIMCD-3 cells (murine epithelial cells from the renal inner medullary collecting duct, CRL-2123, American Type Culture Collection (Rauchman et al., 1993)) were cultured on beads that were not attached to the recording chamber. By applying suction to a recording pipette, a single primary cilium could be made to enter the pipette. If the resistance between the membrane and the pipette was sufficient (≥1 GΩ, or 0.5 GΩ with the low-Ca^2+^ external solution), the cilium was excised from the cell. This left the cilium inside the recording pipette in the inside-out configuration. The pipette containing the cilium was then transferred among different solutions that bathed the cytoplasmic face of the membrane. A schematic diagram appears elsewhere (Kleene and Kleene 2017). Recordings were done under voltage clamp using equipment and software as previously described (Kleene and Kleene 2012). The temperature of the recording chamber was not controlled other than by the room thermostat (24°C). During acquisition, currents were low-pass filtered at 2 kHz and digitized at 5 kHz. For figures, and for analysis of single TRPV4 channels, recordings were again low-pass filtered at 500 Hz. With the exception of the TRPM4 experiments, a correction of 2.7 mV was applied for a liquid junction potential between each internal bath (which contained mostly KCl) and its salt bridge (which contained mostly NaCl). All potentials are given as membrane potential (i.e. internal relative to external).

The beads coated with cells were stored in a standard external (extracellular) solution containing (in mM) NaCl 140, KCl 5, CaCl_2_ 2, MgCl_2_ 2, sodium pyruvate 2, HEPES 5, and D-glucose 9.4, adjusted to pH 7.4 with NaOH. The recording pipettes were filled with this same external solution with two exceptions. In testing the effect of low external Ca^2+^, total CaCl_2_ was reduced to 0.8 mM, and 2 mM BAPTA was added, giving 0.1 µM free Ca^2+^. When studying TRPM4 current, the standard external solution was modified to include KCl 145 and NaCl 0 to maximize the TRPM4 current (Flannery et al., 2015). All internal (cytoplasmic) solutions included KCl 140, NaCl 5, MgCl_2_ 2, HEPES 5, and D-glucose 5, adjusted to pH 7.4 with KOH. Each internal solution also included CaCl_2_ and a Ca^2+^ buffer as described below. U-73122, an inhibitor of phospholipase C (5 µM), and GSK1016790A, a TRPV4 agonist (50 nM) were added to some internal solutions as noted.

For some studies, I selected cilia with no detectable PC2 channels. To check for the presence of TRPP2 channels, the cilium was exposed to one of the solutions with reduced Ca^2+^ buffering. Voltage was maintained at +60 mV. If no large-conductance channels were seen to open within 2 min, active TRPP2 channels were judged to be absent.

### Determination of Starting Free Ca^2+^ Concentrations in Internal Solutions

Apparent association constants *K'*_Ca_ between Ca^2+^ and each of the three Ca^2+^ buffers used were determined by Scatchard analysis (Bers 1982) with a Ca^2+^-selective electrode (Orion 932000). The *K'*_Ca_ values were 6.7 × 10^6^ M^−1^ for BAPTA, 2.0 × 10^6^ M^−1^ for EGTA, and 0.78 × 10^6^ M^−1^ for dibromoBAPTA. The *x*-intercept of the Scatchard plot also yielded the purity of the chelator, which was 84–88%. The impurity is reportedly water (Harrison and Bers 1987; Marks and Maxfield 1991). Calcium chloride solutions were prepared by dilution from a standard 0.1 M solution (Orion 922006) traceable to the National Institute of Standards and Technology. Table 1 lists the buffered Ca^2+^ solutions prepared. Our double-distilled water contained 9 ± 1 µM Ca^2+^ (*n* = 6 determinations), and this is included in the values shown for [Ca^2+^]_total_. The low-Ca^2+^ solutions buffered with BAPTA or EGTA were not sensitive to small errors in the concentrations of total Ca^2+^ or buffer. In all cases, a 2% error (high or low) in either quantity gave a concentration of free Ca^2+^ between 13 and 21 nM.

**TABLE 1:** Ca^2+^ buffering in the internal solutions used.

Ca^2+^ Buffer	[buffer]_total_ (mM)	[Ca^2+^]_total_ (µM)	[Ca^2+^]_free_ (nM)	Figures
BAPTA	0.1	9	15	1, 2, 5, 6
BAPTA	0.25	29	20	3, 4, 7, 8, 9
BAPTA	2	244	21	1, 2, 5, 8
EGTA	0.25	9	19	3, 4
dibromoBAPTA	2	1789	10300	6

### Materials

BAPTA, EGTA, GSK1016790A, and U-73122 were purchased from MilliporeSigma and dibromoBAPTA from Santa Cruz Biotechnology. GSK1016790A was diluted from a 100 µM stock solution and U-73122 from a 5 mM stock solution, both in DMSO.

### Data Analyses

For voltage jumps, the cilium was clamped at a prepulse potential for 10 s, which was then stepped to a more positive (postpulse) potential for 10 s. Prepulse potentials tested ranged from −140 mV to +20 mV and postpulse potentials from +20 mV to +80 mV. For analysis, the mean current in the absence of detectable channel openings (leak current) was subtracted in QuB (State University of New York, Buffalo, NY). Transient currents are defined as the mean current during the first 3 s of the postpulse minus the mean current during the final 3 s of the postpulse. For steady-state currents, mean currents over a 20 s recording, minus the leak current, are reported.

For macroscopic TRPV4 current-voltage relations, current was measured as voltage was continuously varied from −100 or −140 mV to +100 mV. Current at −90 mV was figured as the mean of the values from −100 mV to −80 mV, and current at +90 mV as the mean of the values from +80 mV to +100 mV. For the single-channel study of TRPV4, 20 s recordings were made at each voltage. Single-channel currents were determined from amplitude histograms.

### Statistics

Results are expressed as mean ± standard error. Data sets were compared with a nonparametric test, either the Wilcoxon signed rank test (for paired measures) or the Mann-Whitney rank sum test (for unpaired measures). Our criterion for significance was *p* < 0.05.

## Results

One can predict that there are conditions under which Ca^2+^ entering the cilium through PC2 channels might accumulate inside the cilium to a concentration sufficient to further activate those same PC2 channels. I have been able to verify the existence of such conditions in the laboratory.

### With Reduced Ca^2+^ Buffering, Voltage Prepulses Transiently Activate PC2

**FIGURE 1:**
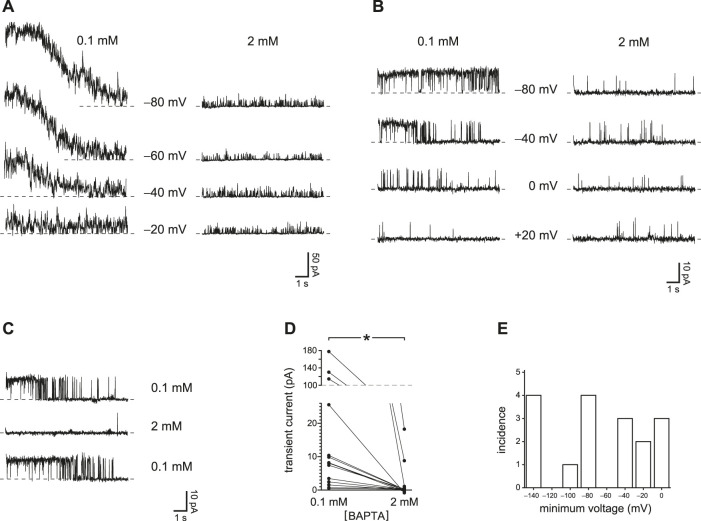
Transient PC2 currents are greater with reduced internal Ca^2+^ buffering (0.1 mM BAPTA compared to 2 mM BAPTA). **(A)** The recordings shown were made with 0.1 mM BAPTA (left) or 2 mM BAPTA (right) at +60 mV immediately following prepulses of 10 s to the voltages shown. Postpulse currents are shown and were measured in the same cilium. This cilium had multiple active PC2 channels, preventing resolution of single-channel events. In this and other recordings, the dashed line represents the current when all PC2 channels were closed. **(B)** In a cilium with just one active PC2 channel, the channel was also activated by prepulses of 10 s to the voltages shown, and again only at the lower concentration of BAPTA. The postpulse voltage was +60 mV. **(C)** The effect of reduced Ca^2+^ buffering is reversible. A cilium with a single PC2 channel showed transient currents in 0.1 mM BAPTA before and after exposure to 2 mM BAPTA, in which transient currents were not seen. Prepulse and postpulse voltages were −70 mV and +40 mV, respectively. **(D)** Paired transient current amplitudes for 15 cilia, each tested in both 0.1 and 2 mM BAPTA. Voltages were chosen that gave the greatest transient current in 0.1 mM BAPTA. Prepulse voltages ranged from −140 mV to −40 mV and postpulse voltages from +40 mV to +80 mV. For a given cilium, the same pair of voltages was used in both 0.1 and 2 mM BAPTA. *p < 0.001. **(E)** Histogram showing the most positive prepulse voltage sufficient to cause a transient current in 0.1 mM BAPTA in each of 17 cilia.

In the primary cilium of mIMCD-3 cells, in the absence of agonists, PC2 channels are the only large-conductance channel seen, and the only channel activated by micromolar concentrations of intraciliary Ca^2+^ (Kleene and Kleene 2017; Liu et al., 2018). Under certain conditions, it was possible to transiently activate the channels to a level above that seen in the steady state. When the cilium was held at a more negative potential for 10 s and then jumped to a more positive potential, such a transient activation could be observed given reduced internal Ca^2+^ buffering (Figure 1A, left, 0.1 mM BAPTA; Supplementary Figure S1). The transient activity was greater when the prepulse potential was more negative. The internal solution for these tests was prepared to have a free Ca^2+^ concentration of 15 nM, and the external solution included 2 mM Ca^2+^. Transferring the cilium to an internal solution with a greater buffer concentration (2 mM), while maintaining the low free Ca^2+^ concentration, prevented the transient current (Figure 1A, right, 2 mM BAPTA). This indicates that the transient activity results from an increase in intraciliary free Ca^2+^ that can be overcome with sufficient Ca^2+^ buffering. Although there is a driving force for Ca^2+^ to enter the cilium at all of the voltages tested, the prepulse temporarily increases that driving force. Under favorable conditions, even a single PC2 channel might admit enough Ca^2+^ to further activate itself. Cilia with a single active PC2 channel are encountered on occasion. In each of 4 such cases tested, the channel showed a transient activation following a prepulse to a sufficiently negative potential if Ca^2+^ buffering was low (Figure 1B, left). Again, the effect was not seen with increased Ca^2+^ buffering (Figure 1B, right). The reduction of activity with higher Ca^2+^ buffering was reversible (Figure 1C). In each of 17 cilia tested in 0.1 mM BAPTA, a prepulse protocol could be found that led to a transient activation given reduced Ca^2+^ buffering. In 15 cilia tested in both 0.1 and 2 mM BAPTA, the maximum mean transient current was 34 ± 15 pA with 0.1 mM BAPTA and 1.9 ± 1.3 pA with 2 mM BAPTA (Figure 1D). The difference is significant (*p* < 0.001, Wilcoxon signed rank test for paired measures). Prepulse voltages sufficient to cause transient activation varied from one cilium to the next. Figure 1E shows the most positive prepulse potential able to cause transient activation in 0.1 mM BAPTA.

### With Reduced Ca^2+^ Buffering, Steady-State PC2 Activity Also Increases

**FIGURE 2:**
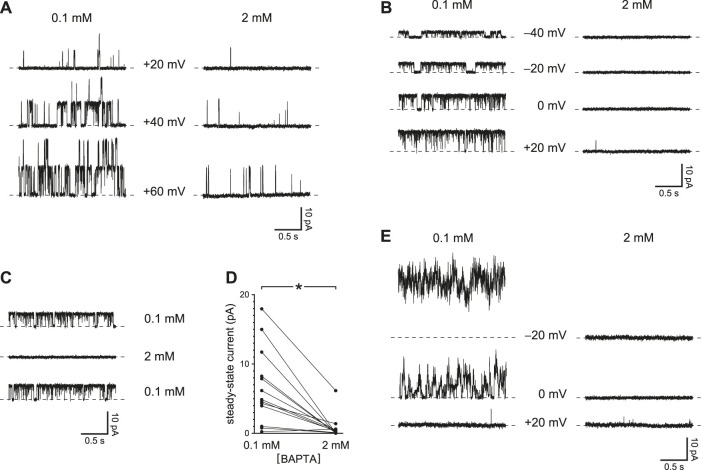
Steady-state activity of PC2 channels is greater with reduced Ca^2+^ buffering (0.1 mM BAPTA compared to 2 mM BAPTA). **(A)** Steady-state activity was measured in one cilium at the voltages shown in 0.1 mM BAPTA (left) and 2 mM BAPTA (right). **(B)** In a cilium with just one PC2 channel, the channel also showed higher steady-state activity with the lower concentration of BAPTA. **(C)** The effect of reduced Ca^2+^ buffering is reversible. A cilium with a single PC2 channel showed higher steady-state activity in 0.1 mM BAPTA before and after exposure to 2 mM BAPTA. Voltage was maintained at 0 mV. **(D)** Paired mean steady-state currents for 13 cilia, each tested in both 0.1 and 2 mM BAPTA. Voltages were chosen that gave the greatest difference in mean current between 0.1 and 2 mM BAPTA and ranged from −20 to +80 mV *p < 0.001. **(E)** Steady-state currents in the one cilium in which activity increased with hyperpolarization in the presence of 0.1 mM BAPTA.

The voltage prepulses I tested temporarily increased the driving force for Ca^2+^ to enter the cilium. Even at a steady potential, though, PC2 channels might conduct enough Ca^2+^ into the cilium to increase their own activities. This was also confirmed experimentally. In 12 of 13 cilia tested, channel activity over some range of holding potentials was greater when Ca^2+^ buffering was reduced (Figure 2A, left) than with higher Ca^2+^ buffering (Figure 2A, right). The effect was observed even in each of 3 cilia with just one active PC2 channel (Figure 2B) and was reversible (Figure 2C). In the 13 cilia tested, the mean steady-state current was 6.7 ± 1.5 pA with 0.1 mM BAPTA and 0.8 ± 0.5 pA with 2 mM BAPTA (Figure 2D). The difference is significant (*p* < 0.001, Wilcoxon signed rank test for paired measures).

In all but one cilium, the activity of the channel showed a voltage dependence that has been well described, increasing as the steady-state potential was made more positive. In a single cilium, however, channel activity decreased with low Ca^2+^ buffering as the potential was changed from −20 to +20 mV (Figure 2E). This sequence was repeated 3 times in this cilium with the same result.

### Postpulse and Steady-State Activity are Greater When a Slower Ca^2+^ Buffer is Used

**FIGURE 3:**
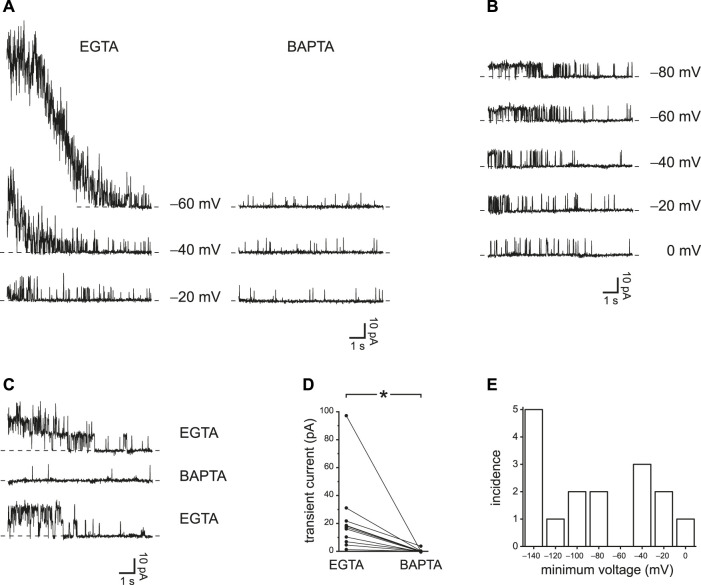
Transient PC2 currents are greater with slower Ca^2+^ buffering (0.25 mM EGTA compared to 0.25 mM BAPTA). **(A)** The recordings shown were made with 0.25 mM EGTA (left) or 0.25 mM BAPTA (right) at +20 mV immediately following prepulses of 10 s to the voltages shown. Postpulse currents are shown and were measured in the same cilium. This cilium had multiple active PC2 channels, preventing resolution of single-channel events. **(B)** In a cilium with just one PC2 channel, transient currents were also activated by prepulses of 10 s to the voltages shown. The postpulse voltage was +40 mV and the buffer was 0.25 mM EGTA. **(C)** The effect of slower Ca^2+^ buffering is reversible. A cilium showed transient currents in 0.25 mM EGTA before and after exposure to 0.25 mM BAPTA, in which transient currents were not seen. Prepulse and postpulse voltages were −80 mV and +60 mV, respectively. **(D)** Paired transient current amplitudes for 11 cilia, each tested in both 0.25 mM EGTA and 0.25 mM BAPTA. Voltages were chosen that gave the greatest transient current in 0.25 mM EGTA. Prepulse voltages ranged from −140 to −60 mV and postpulse voltages from +20 to +80 mV. For a given cilium, the same pair of voltages was used in both EGTA and BAPTA. *p < 0.001. **(E)** Histogram showing the most positive prepulse voltage sufficient to cause a transient current in 0.25 mM EGTA in each of 16 cilia.

EGTA should be less effective than BAPTA at restricting the accumulation of intraciliary Ca^2+^. Although the two buffers have similar equilibrium association constants with Ca^2+^ at cellular pH, the rate of Ca^2+^ binding by BAPTA is roughly 100 times faster than the rate for EGTA (Dargan and Parker 2003). Thus a sufficiently rapid influx of Ca^2+^ may overcome buffering by EGTA but not by BAPTA. I have confirmed this prediction by comparing the effects of the two Ca^2+^ buffers. With 0.25 mM EGTA as the buffer in the internal solution, it was possible in each of 11 cilia tested to identify a prepulse potential that allowed a transient activation of PC2 channels after jumping to a more positive potential (Figure 3A, left, EGTA; Supplementary Figure S2). The transient activation was absent when the cilia were transferred to an internal solution with 0.25 mM BAPTA instead of EGTA (Figure 3A, right, BAPTA). The internal solutions with both EGTA and BAPTA were prepared to have free Ca^2+^ concentrations of about 20 nM. With EGTA, the transient activation was seen in each of 4 cilia tested that had a single active PC2 channel (Figure 3B), and the effect of BAPTA was reversible (Figure 3C). In 11 cilia tested in both EGTA and BAPTA (each at 0.25 mM), the maximum mean transient current was reduced from 22 ± 8 pA with EGTA to 0.3 ± 0.3 pA with BAPTA (Figure 3D). The effect of buffer choice is significant (*p* < 0.001, Wilcoxon signed rank test for paired measures). Figure 3E shows the most positive prepulse potential able to cause transient activation in each of 16 cilia tested in EGTA.

**FIGURE 4:**
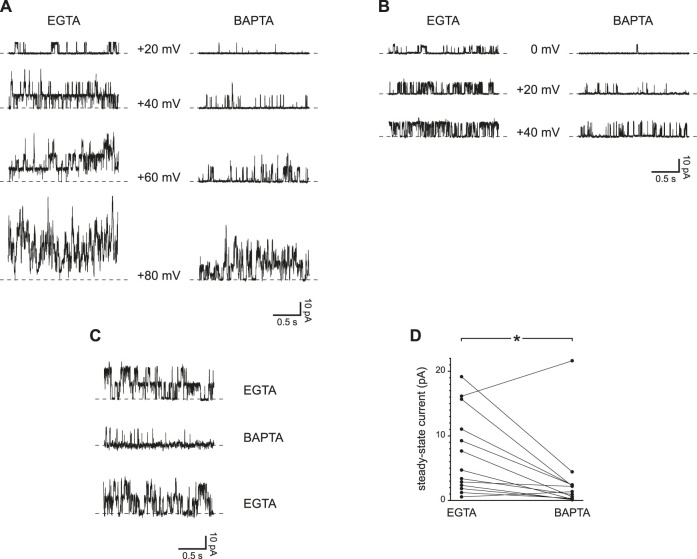
Steady-state activity of PC2 channels is greater with slower Ca^2+^ buffering (0.25 mM EGTA compared to 0.25 mM BAPTA). **(A)** Steady-state activity was measured in one cilium at the voltages shown in 0.25 mM EGTA (left) and 0.25 mM BAPTA (right). **(B)** In a cilium with just one PC2 channel, the channel also showed higher steady-state activity in 0.25 mM EGTA than in 0.25 mM BAPTA. **(C)** The effect of slower Ca^2+^ buffering is reversible. A cilium showed higher steady-state activity in 0.25 mM EGTA before and after exposure to 0.25 mM BAPTA. Voltage was maintained at +60 mV. **(D)** Paired mean steady-state currents for 13 cilia, each tested in both 0.25 mM EGTA and 0.25 mM BAPTA. Voltages were chosen that gave the greatest difference in mean current between 0.25 mM EGTA and 0.25 mM BAPTA and were in all cases +60 or +80 mV *p = 0.010.

In most cilia tested, steady-state channel activity was greater at some holding potentials with EGTA as the Ca^2+^ buffer instead of BAPTA (each at 0.25 mM). This was seen in cilia with several PC2 channels (Figure 4A) and in 3 cilia each with a single channel (Figure 4B). The effect of exposure to BAPTA was reversible (Figure 4C). In the 13 cilia tested, the mean steady-state current was 7.4 ± 1.8 pA with 0.25 mM EGTA and 3.0 ± 1.6 pA with 0.25 mM BAPTA (Figure 4D). The difference is significant (*p* = 0.010, Wilcoxon signed rank test for paired measures).

### The Effects of Reduced Ca^2+^ Buffering Require External Ca^2+^

**FIGURE 5:**
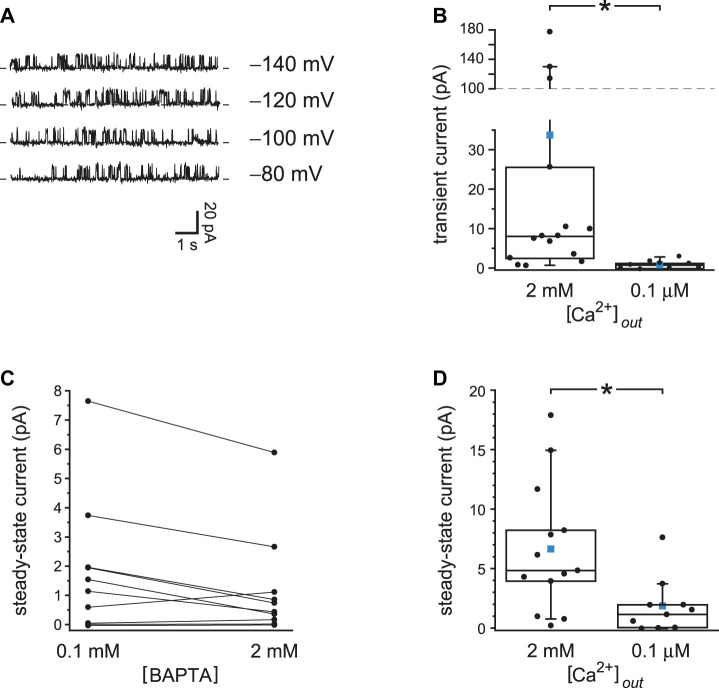
With reduced external Ca^2+^, PC2 current is insensitive to Ca^2+^ buffering. **(A)** The recordings shown were made at +60 mV immediately following a prepulse of 10 s to the voltages shown. The internal solution contained 0.1 mM BAPTA and 15 nM free Ca^2+^ and the external solution 0.1 µM free Ca^2+^. **(B)** Transient current amplitudes for cilia tested with either 2 mM (*n* = 15 cilia) or 0.1 µM (*n* = 9 cilia) external free Ca^2+^; the internal solution contained 0.1 mM BAPTA. Voltages were chosen that gave the greatest transient current. Prepulse voltages ranged from −140 mV to −40 mV and postpulse voltages from +40 mV to +80 mV. The values for cilia in 2 mM external Ca^2+^ are taken from Figure 1D. *p < 0.001. **(C)** Paired mean steady-state currents for 10 cilia, each tested in both 0.1 and 2 mM internal BAPTA; the external solution contained 0.1 µM free Ca^2+^. Voltages were chosen that gave the greatest difference in mean current between 0.1 and 2 mM BAPTA and were either +40 or +60 mV. **(D)** Mean steady-state currents for cilia tested with either 2 mM (*n* = 13 cilia) or 0.1 µM (*n* = 10 cilia) external free Ca^2+^; the internal solution contained 0.1 mM BAPTA. The values for cilia in 2 mM and 0.1 µM external Ca^2+^ are taken from Figure 2D and Figure 5C, respectively. In the box plots, the individual data are shown as filled circles. The top, middle, and bottom lines indicate the 75th, 50th, and 25th percentile values, respectively. Whiskers indicate the 90th and 10th percentile values. A blue square is shown at the level of the mean value. *p = 0.0099.

Each experiment shown so far compares two internal solutions that were prepared to have identical concentrations of free Ca^2+^, in all cases well below the ∼1 µM or more needed to activate PC2 in previous reports (Kleene and Kleene 2017; Liu et al., 2018). Voltage protocols were found that allowed activation of PC2 channels to levels expected for much higher intraciliary Ca^2+^ concentrations than the concentrations initially provided. The dependence of this activation on the concentrations and rate constants of the Ca^2+^ buffers shows that the activation is Ca^2+^-dependent. Apparently there is a source of intraciliary Ca^2+^ beyond that present in the internal solutions provided at the start. To test whether the source is external Ca^2+^ that crosses the membrane to the intraciliary compartment, the experiments were repeated with external Ca^2+^ reduced from 2 mM to 0.1 µM. With reduced external Ca^2+^, voltage prepulses even to −140 mV caused no transient activation of PC2 (Figure 5A). With 2 mM external Ca^2+^, the maximum mean transient current following a prepulse averaged 34 ± 15 pA (*n* = 15 cilia); with 0.1 µM external Ca^2+^ the value was 0.8 ± 0.4 pA (*n* = 9 cilia) (Figure 5B). The values are significantly different (*p* < 0.001, Mann-Whitney rank sum test for unpaired measures). This was true (*p* = 0.0024) even if the three very high values from the population tested in 2 mM Ca^2+^ were excluded. With reduced external Ca^2+^, steady-state PC2 current at +40 or +60 mV did not depend on the concentration of the Ca^2+^ buffer (Figure 5C). The mean currents averaged 1.9 ± 0.7 pA with 0.1 mM BAPTA and 1.2 ± 0.6 pA with 2 mM BAPTA. The difference is not significant (*n* = 10 cilia, *p* = 0.084, Wilcoxon signed rank test for paired measures). With reduced cytoplasmic Ca^2+^ buffering, mean steady-state current was greater with 2 mM external Ca^2+^ (6.7 ± 1.5 pA, *n* = 13 cilia) than with 0.1 µM external Ca^2+^ (1.9 ± 0.7 pA, *n* = 13 cilia) (Figure 5D). The difference is significant (*p* = 0.0099, Mann-Whitney rank sum test for unpaired measures).

### Reduced Ca^2+^ Buffering Does Not Affect the Activity of TRPM4 Channels, Which Are Activated by Ca^2+^ but do not Conduct it

The additional Ca^2+^ that activates PC2 channels when Ca^2+^ buffering is reduced may enter through the PC2 channels themselves, but the experiments thus far do not allow that conclusion. An ideal control would be a PC2 channel that has the usual sensitivity to intraciliary Ca^2+^ but does not conduct Ca^2+^. If transient activation were to be seen with such a channel, it would indicate that there is a Ca^2+^ source other than flow through PC2 itself. No such PC2 variant has been described, but the renal primary cilium has another channel that can serve this purpose: TRPM4. These channels are present in the primary cilia of most mIMCD-3 cells and, like other TRPM4 channels reported, are activated by internal Ca^2+^ but do not conduct Ca^2+^ (Mathar et al., 2014; Flannery et al., 2015). In typical experimental conditions, including those used above, they are much less sensitive than PC2 to internal Ca^2+^. When phospholipase C is inhibited, though, TRPM4, like PC2, is activated by micromolar levels of internal Ca^2+^ (Zhang et al., 2005; Nilius et al., 2006; Flannery et al., 2015). Many of the renal primary cilia lack active PC2 channels (Kleene and Kleene 2017; Ha et al., 2020), and such cilia were chosen to study TRPM4 currents in isolation. In these cilia, I inhibited phospholipase C by adding 5 µM U-73122 to the internal solutions and looked for a source of Ca^2+^ influx that might activate TRPM4 channels.

**FIGURE 6:**
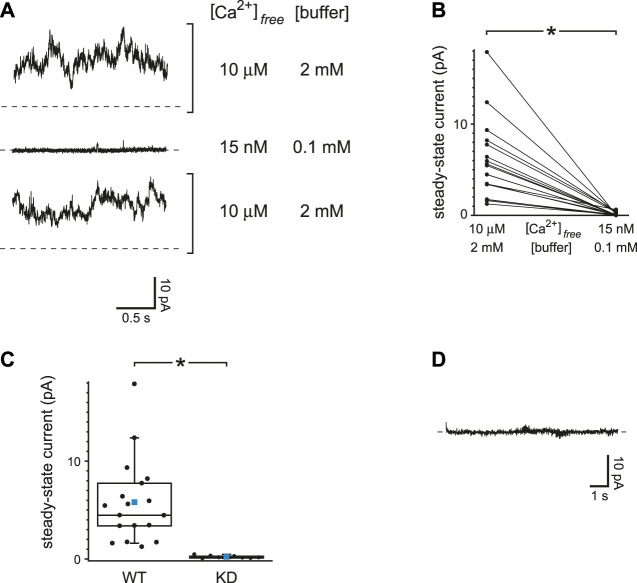
TRPM4 current is insensitive to Ca^2+^ buffering. **(A)** Steady-state activity was measured at +60 mV in each of two internal solutions. The first of these (top and bottom recordings) had 10 µM free Ca^2+^ buffered with 2 mM dibromoBAPTA. The other solution had 15 nM free Ca^2+^ buffered with 0.1 mM BAPTA. **(B)** Paired mean steady-state currents at +60 mV for 18 cilia, each tested in each of the two internal solutions. *p < 0.001. **(C)** Mean steady-state currents at +60 mV in 10 µM free Ca^2+^ buffered with 2 mM dibromoBAPTA in cilia from wild-type cells (WT, *n* = 18 cilia) or cells in which TRPM4 was knocked down with shRNA (KD, *n* = 8 cilia). The values for wild-type cells are taken from Figure 6B. *p < 0.001. **(D)** Absence of transient activation of TRPM4 in the internal solution containing 15 nM free Ca^2+^ buffered with 0.1 mM BAPTA. The current shown was measured at +60 mV immediately following a prepulse of 10 s to −140 mV. Recordings in parts A and D are from the same cilium.

The top trace of Figure 6A shows the steady-state TRPM4 current activated at +60 mV when internal free Ca^2+^ was buffered to 10 µM with 2 mM dibromoBAPTA. Under this condition a macroscopic TRPM4 current was activated that averaged 5.8 ± 1.0 pA at +60 mV (*n* = 18 cilia). When a cilium was moved to an internal solution with just 15 nM free Ca^2+^ but also reduced Ca^2+^ buffering (0.1 mM BAPTA), almost no TRPM4 current was seen (Figure 6A, middle recording). In this case the mean TRPM4 current averaged 0.14 ± 0.04 pA (*n* = 18 cilia). The reduction of TRPM4 current is significant (Figure 6B, *n* = 18 cilia, *p* < 0.001, Wilcoxon signed rank test for paired measures). On return to the internal solution with 10 µM free Ca^2+^, the TRPM4 current was restored (Figure 6A, bottom trace). On average, the ratio of the TRPM4 current seen with 15 nM Ca^2+^ and reduced buffering to that activated in 10 µM free Ca^2+^ was 0.03 ± 0.01 (*n* = 18 cilia).

To verify that the Ca^2+^-activated current was conducted through TRPM4 channels, the experiment was repeated in cells in which TRPM4 was knocked down with shRNA (Flannery et al., 2015). In cilia from such cells, the steady-state current activated by 10 µM free Ca^2+^ was 0.21 ± 0.05 pA (*n* = 8 cilia), which is significantly less that the current in wild-type cells (Figure 6C, *p* < 0.001, Mann-Whitney rank sum test for unpaired measures). With reduced Ca^2+^ buffering, a prepulse to a negative voltage did not give rise to a detectable transient TRPM4 current in cilia from wild-type cells when the voltage was jumped from −120 or −140 mV to +60 mV (Figure 6D). Following a prepulse, the mean transient current was 0.15 ± 0.12 pA (*n* = 10 cilia). In the absence of PC2 channels, I found no evidence of a Ca^2+^ influx that could activate TRPM4 even with reduced Ca^2+^ buffering. This supports the hypothesis that the activation of PC2 described above occurs following an influx of Ca^2+^ through the PC2 channels themselves.

### Reduced Ca^2+^ Buffering Increases the Activity of TRPV4 Channels, Which, Like PC2, are Activated by Ca^2+^ and Conduct Ca^2+^

**FIGURE 7:**
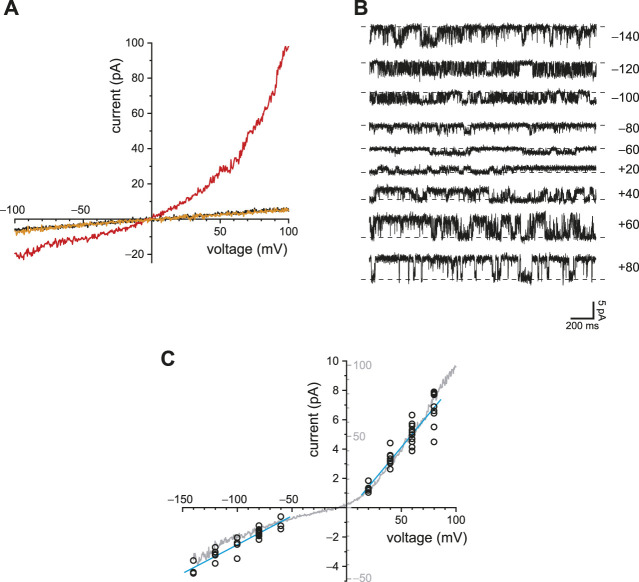
Ciliary TRPV4 activity. **(A)** In a single cilium, the ciliary current-voltage relation was measured in the absence of the TRPV4 agonist GSK1016790A (black), in its presence (50 nM, red), and again in its absence (orange). **(B)** Single-channel current fluctuations observed in the presence of 50 nM GSK1016790A at the membrane potentials shown. **(C)** Single-channel current-voltage relation from −140 to +80 mV (open circles). The internal solution contained 50 nM GSK1016790A. At each voltage, single-channel current was measured in from 3 to 11 cilia. The blue lines show the best linear fits and have slopes (single-channel conductances) of 39 pS (−140 to −60 mV) and 90 pS (+20 to +80 mV). The relation shown in gray is the average of the macroscopic currents activated by 50 nM GSK1016790A in each of 8 cilia. Leak currents measured in the absence of GSK1016790A were subtracted. The current scales of the single-channel and macroscopic currents are different and are both shown on the *y*-axis. The scales were chosen to facilitate comparison of the single-channel and macroscopic current-voltage relations. In all parts of this figure, Ca^2+^ was buffered with 0.25 mM BAPTA.

The primary cilia of mIMCD-3 cells also express TRPV4 channels. To study TRPV4 currents in isolation, cilia lacking active PC2 channels were again selected. With internal Ca^2+^ buffered with 0.25 mM BAPTA, the ciliary macroscopic current-voltage relation showed a small leak conductance (Figure 7A, black). Addition of the TRPV4 agonist GSK1016790A (50 nM; Willette et al., 2008) activated an outwardly rectifying macroscopic current (Figure 7A, red). The activation was reversible on removal of the agonist (Figure 7A, orange). With 0.25 mM BAPTA, the additional conductance seen in the presence of GSK1016790A averaged 1,648 ± 449 pS measured between +20 mV and +80 mV (*n* = 18 cilia). The mean reversal potential of the additional current (intersection of the red and black recordings) was −9.0 ± 0.6 mV (*n* = 14 cilia).

In a few cilia, the number of active channels was low enough to allow resolution of single-channel events (Figure 7B). The average single-channel conductance was 39 pS when measured between −140 and −60 mV and 90 pS between +20 and +80 mV (Figure 7C, blue lines). The current-voltage relation of the macroscopic current (Figure 7C, gray) was similar to that of the single channels, suggesting that the channel open probability does not strongly depend on voltage. The relief of outward rectification at very negative potentials is characteristic of TRPV4 in the presence of extracellular Ca^2+^ (Voets et al., 2002).

**FIGURE 8:**
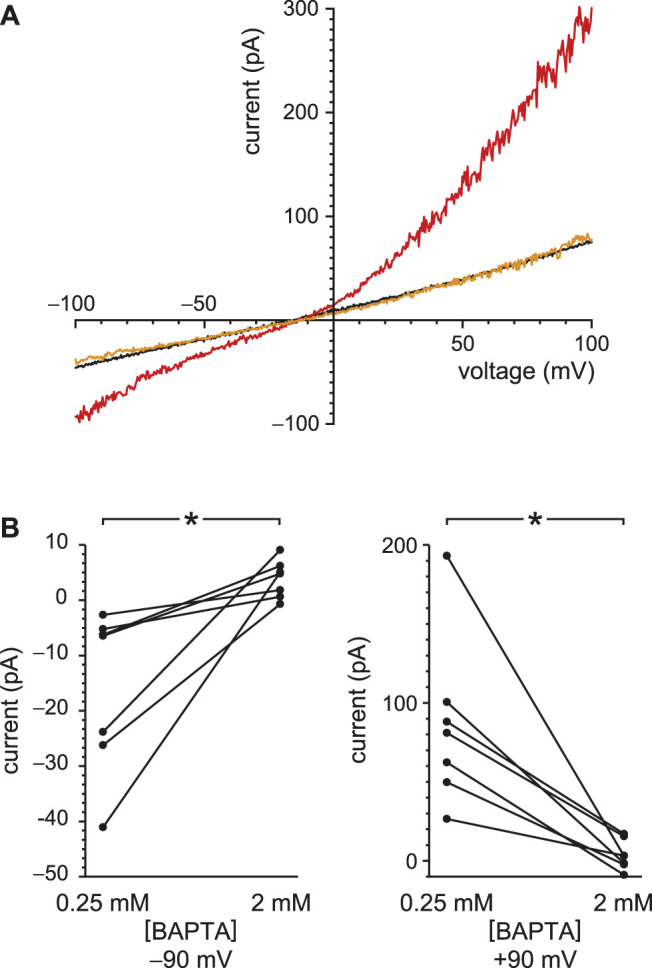
Steady-state TRPV4 activity is greater with reduced Ca^2+^ buffering (0.25 mM BAPTA compared to 2 mM BAPTA). **(A)** In a single cilium, the current-voltage relation was measured with 0.25 mM BAPTA (black), in the same solution after addition of the TRPV4 agonist GSK1016790A (50 nM, red), and in a solution with 50 nM GSK1016790A but with BAPTA increased to 2 mM (orange). **(B)** Paired currents activated by 50 nM GSK1016790A in 10 cilia, each tested in both 0.25 mM BAPTA and 2 mM BAPTA. The left plot shows current at −90 mV and the right at +90 mV *p = 0.0016 at both potentials.

TRPV4 channels can be activated by internal Ca^2+^ and also conduct Ca^2+^ (Strotmann et al., 2003). In other systems, this influx potentiates their activity by positive feedback, producing a regenerative response (Strotmann et al., 2003; White et al., 2016). I was able to demonstrate this effect in renal primary cilia. With reduced buffering of internal Ca^2+^ (0.25 mM BAPTA), 50 nM internal GSK1016790A activated an outwardly rectifying current (Figure 8A, compare black and red). When the internal solution was replaced with one still containing 50 nM GSK1016790A but with BAPTA increased to 2 mM (Figure 8A, orange), the agonist-activated current was greatly reduced. With 0.25 mM BAPTA, the additional current seen in the presence of GSK1016790A averaged −16 ± 6 pA at −90 mV and 86 ± 20 pA at +90 mV (Figure 8B, *n* = 7 cilia). With 2 mM BAPTA, the additional current averaged 3.8 ± 1.3 pA at −90 mV and 3.7 ± 3.6 pA at +90 mV (Figure 8B, *n* = 7 cilia). The effect of buffer concentration is significant at both potentials (*p* = 0.0016 at either potential, Wilcoxon signed rank test for paired measures).

### With Reduced Ca^2+^ Buffering, Ca^2+^ Influx Through TRPV4 can Secondarily Activate PC2

**FIGURE 9:**
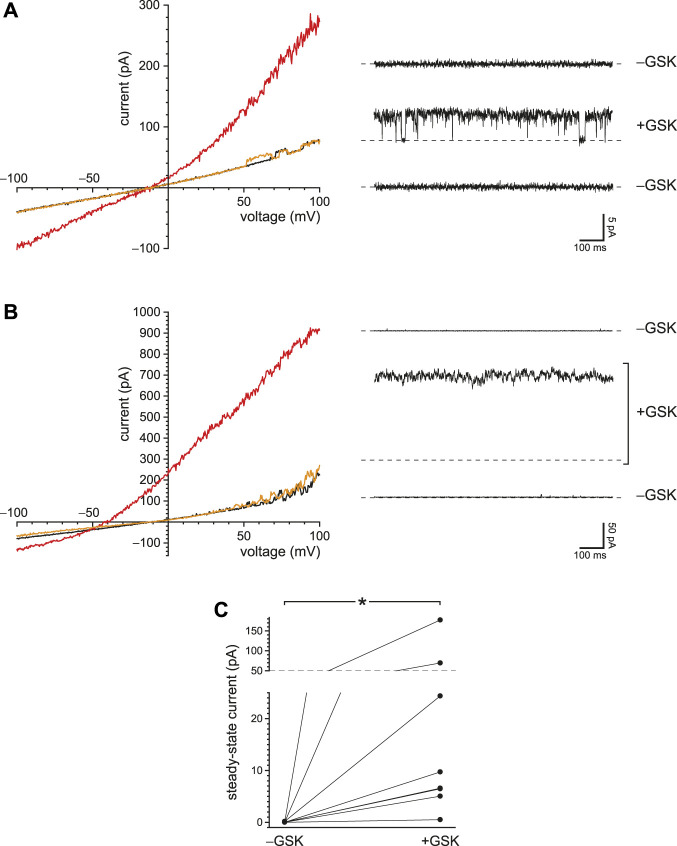
Activation of PC2 secondarily to a Ca^2+^ influx through TRPV4 channels. **(A)** In a single cilium with just one active PC2 channel, the ciliary current-voltage relation (left) was measured in the absence of the TRPV4 agonist GSK1016790A (black), in its presence (50 nM, red), and again in its absence (orange). Steady-state PC2 activity at −5 mV was reversibly activated by GSK1016790A (right). For each of the three recordings, a dashed line is shown at the level of the leak current (3.6 pA). The same cilium was used for the left and right figures. **(B)** A repeat of the experiment shown in **(A)** but using a cilium with multiple PC2 channels. The dashed lines again show the leak current (4.7 pA). **(C)** Paired mean PC2 currents at −5 mV after subtraction of leak currents in 8 cilia, each tested in the absence and presence of GSK1016790A. In all parts of this figure, Ca^2+^ was buffered with 0.25 mM BAPTA. *p = 0.0078.

Both PC2 and TRPV4 conduct Ca^2+^ and can be activated by intraciliary Ca^2+^. Thus a Ca^2+^ influx through one of these channel types might activate the other. I tested whether ciliary PC2 channels could show a secondary activation following direct activation of TRPV4 in the presence of 0.25 mM BAPTA. Figure 9A shows the outcome in a cilium with just one active PC2 channel. With reduced internal Ca^2+^ and no agonist, the PC2 channel opened only at strongly depolarizing potentials (Figure 9A, left, black), as expected (Kleene and Kleene 2017; Liu et al., 2018). Addition of the TRPV4 agonist GSK1016790A (50 nM) increased the current at most potentials (Figure 9A, left, red). In general, it is not straightforward to assess the proportions of the PC2 and TRPV4 currents after addition of the agonist. GSK1016790A activates a TRPV4 current with a reversal potential near −9.0 mV (Figure 7A); the reversal potential of PC2 under these conditions is −61 mV (Kleene and Kleene 2017). The mixed current activated by GSK1016790A reversed at −13 mV (Figure 9A, left, intersection of the red and black recordings), suggesting that it was mostly carried by TRPV4 channels. Near the reversal potential for TRPV4 (−9.0 mV), there is no net TRPV4 current, so one can measure the PC2 current in isolation. (Note that even at this potential there is still an inward driving force for Ca^2+^. The influx of Ca^2+^ and Na^+^ is cancelled by an efflux of K^+^.) On addition of 50 nM GSK1016790A, the open probability of the single PC2 channel at −5 mV increased from almost 0 to a value approaching 1 (Figure 9A, right). On return to a solution lacking GSK1016790A, the current at all potentials returned to the lower level (Figure 9A, left, orange), and the single PC2 channel returned to an open probability near 0 (Figure 9A, right, bottom).

In a second cilium, the agonist activated a mean PC2 current at −5 mV of 177 pA (Figure 9B, right), which equals the current expected from 32 PC2 channels, assuming an open probability of 1 (Kleene and Kleene 2017). The current activated by GSK1016790A in this cilium reversed at −52 mV (Figure 9B, left), suggesting that most of the activated current was carried by PC2 channels. Of 8 cilia tested, 7 showed increased PC2 activity at −5 mV in the presence of 50 nM GSK1016790A (Figure 9C). PC2 channels were almost never seen to open at −5 mV in the absence of GSK1016790A; the mean channel current never exceeded 0.17 pA and averaged 0.025 ± 0.021 pA (*n* = 8 cilia). In the presence of GSK1016790A, the mean current in the same cilia at −5 mV averaged 37 ± 21 pA (*n* = 8 cilia). The effect of GSK1016790A on PC2 activity is significant (*p* = 0.0078, Wilcoxon signed rank test for paired measures).

## Discussion

### The Renal Primary Cilium Can Support Regenerative Ca^2+^ Signaling

**FIGURE 10:**
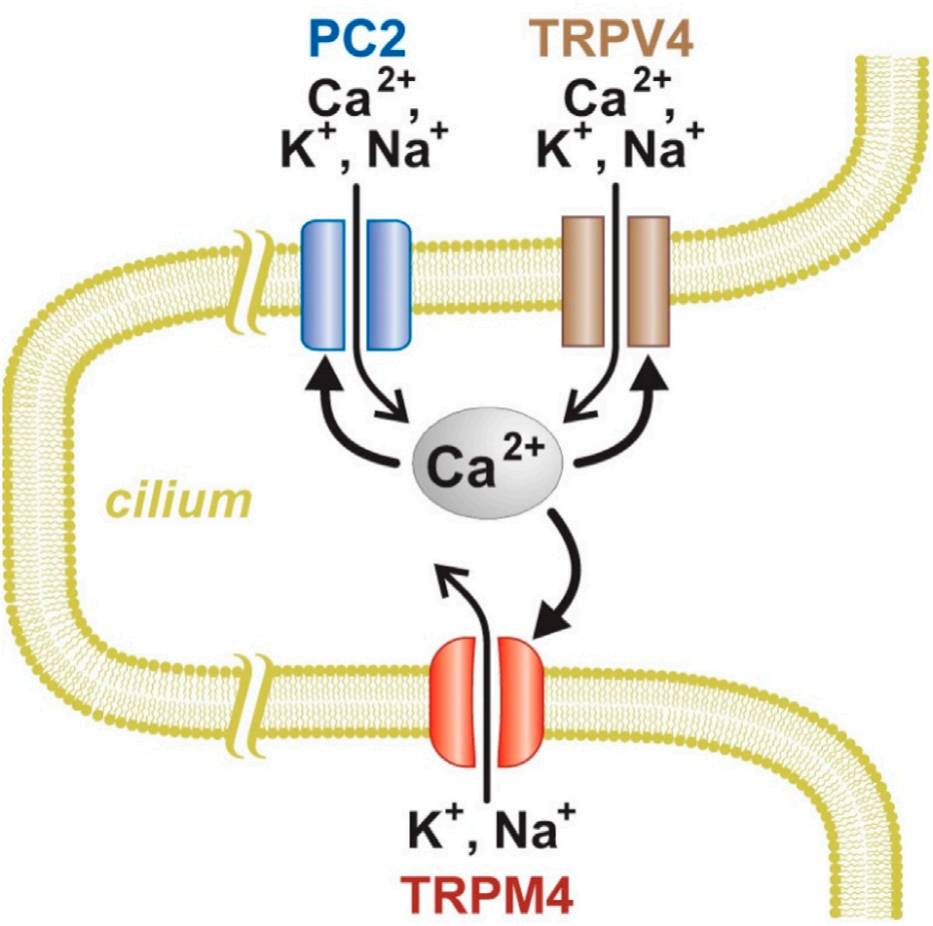
Model of Ca^2+^ signaling and amplification in renal primary cilium. PC2 and TRPV4, but not TRPM4, can conduct Ca^2+^ into the cilium. An increase in intraciliary Ca^2+^ can further activate all three of the channel types.

The work presented here identifies conditions that allow regenerative Ca^2+^ signaling in the primary cilia of renal epithelial cells. Figure 10 summarizes that system. The cilia contain at least two types of ion channels, PC2 and TRPV4, that conduct Ca^2+^ from the external compartment and also are activated by intraciliary Ca^2+^. This can result in positive feedback: As Ca^2+^ enters the cilium, it increases the activity of PC2 and TRPV4 channels, causing further Ca^2+^ entry, which causes further activation, and so on. Intraciliary Ca^2+^ also increases the open probability of TRPM4 channels, but those do not conduct Ca^2+^. Opening any of these channels should depolarize the cilium. That may further contribute to a regenerative response, since the PC2 channels are also activated by depolarization (Kleene and Kleene 2017; Liu et al., 2018). PC2 is also part of a regenerative system in the endoplasmic reticulum of renal cells (Sammels et al., 2010). There PC2 closely interacts with another Ca^2+^-activated, Ca^2+^-conducting channel, the inositol 1,4,5-trisphosphate receptor (IP_3_R). The interaction potentiates Ca^2+^-induced Ca^2+^ release unless the channel function of PC2 is eliminated by mutation. The potentiation is sensitive to the speed and concentrations of the Ca^2+^ buffers chosen. A similar system was described in oocytes overexpressing PC2 and IP_3_R (Li et al., 2005). By contrast, in artificial bilayers, PC2 inhibits the activity of single channels of another Ca^2+^-activated, Ca^2+^-conducting channel, the ryanodine receptor (Anyatonwu et al., 2007).

### Effects of Ca^2+^ Buffers on Ciliary Ca^2+^ Signals

Not surprisingly, the concentrations and speeds of the internal Ca^2+^ buffers profoundly influenced the extent of positive feedback in the cilium. With low internal free Ca^2+^ and 2 mM BAPTA, the open probability of PC2 does not exceed ∼0.2, even at favorable potentials (e.g. +80 mV; Kleene and Kleene 2017). When the initial internal solution contained low Ca^2+^ but just 0.1 mM BAPTA, PC2 channels neared a fully open state even at −40 mV (Figure 2B). This reflects an intraciliary free Ca^2+^ concentration >10 μM, as judged by measurements made with stronger Ca^2+^ buffering (2 mM BAPTA; Kleene and Kleene 2017). This is an increase of at least 500-fold over the free Ca^2+^ initially provided (15–21 nM). The additional intraciliary Ca^2+^ arose from Ca^2+^ flowing through PC2 channels into the cilium, where it was able to overcome the dilute Ca^2+^ buffer and accumulate. Consistent with this explanation, positive feedback could not be achieved with low external Ca^2+^, nor with a channel (TRPM4) that is activated by Ca^2+^ but does not conduct it. When BAPTA was replaced with EGTA, a buffer that is slower to bind Ca^2+^, positive feedback was enhanced (Figures 3, 4). Activation of TRPV4 by an agonist was also dependent on Ca^2+^ buffering; it was successful with 0.25 mM BAPTA but not 2 mM BAPTA (Figure 8). Regenerative signaling by TRPV4 has been described in other systems (Strotmann et al., 2003; White et al., 2016).

### Range of Intraciliary Ca^2+^ Signals

In a limiting case, a single PC2 channel was able to conduct enough Ca^2+^ into the cilium to further activate itself (Figures 1B, C, 2B,C, 3B, 4B). This presumably requires diffusion of Ca^2+^ over a very short distance, from the mouth of the channel pore to an internal Ca^2+^-activation site on the same channel, which has not been identified (Vien et al., 2020). This self-activation was somewhat more effective with EGTA than with BAPTA (Figures 3, 4). In models of other Ca^2+^-conducting channels, internal Ca^2+^ ranges from 50 µM to over 200 μM at the center of the channel (Naraghi and Neher 1997; Neher 1998; Berkefeld et al., 2006; Foskett et al., 2007). The effects of EGTA and BAPTA are expected to differ only over distances of about 20–200 nm from the Ca^2+^ source (Neher 1998; Berkefeld et al., 2006), suggesting that the site of PC2 self-activation by Ca^2+^ is within this distance from the pore. Whether the Ca^2+^ domain of a single channel extends to other channels should depend on the Ca^2+^ buffers and on the distributions of the channels along the length of the cilium. In dendritic segments, Ca^2+^ signals can spread over distances of 5–500 µm (Augustine et al., 2003). Internal Ca^2+^ can be as high as 5–10 µM even 200 nm from the Ca^2+^ source (Neher 1998). This would be more than enough to activate the ciliary channels, but the size of the domain depends strongly on the speed and concentration of the buffer (Berkefeld et al., 2006). In cilia with multiple PC2 channels, the regenerative effects might have resulted strictly from individual channels locally self-activating. However, activation of TRPV4 channels with an agonist increased intraciliary Ca^2+^ enough for secondary activation of PC2 channels (Figure 9). The relative positions of the TRPV4 and PC2 channels are not known.

### Theoretical Considerations for Future Models

It would be instructive to model this system, but one would need to know the identities, concentrations, mobilities, rate constants, and Ca^2+^ affinities of any mobile or immobile intraciliary Ca^2+^ buffers; the locations and Ca^2+^ conductances of the relevant channels; the locations and sensitivities of their Ca^2+^-activation sites; and the diffusion geometry within the cilium. Most of these parameters are not yet available. The cilium does not contain endoplasmic reticulum. There could be membrane transporters that eject Ca^2+^, although none have been described in these cilia. The results presented here suggest some obvious tradeoffs that a model might help to explain. For example, making the voltage more negative increases the driving force for Ca^2+^ entry, which should increase the response. However, it also decreases the open probability of the PC2 channels (Kleene and Kleene 2017; Liu et al., 2018), which should decrease the response. In most cilia, more negative voltage increased the response, suggesting that the driving force for Ca^2+^ dominated. However, a single cilium (Figure 2E) showed the opposite effect of voltage. The voltages needed to optimize the response varied substantially from cilium to cilium (Figures 1E, 3E). A model could reveal how sensitive the response is to variation in each parameter.

### Relevance to the Native Renal Epithelial Cell

I have identified laboratory conditions that strongly potentiate Ca^2+^ signals in the cilium. Those conditions cannot perfectly mimic an undisturbed cell. I recorded at 24°C. At 37°C, the native Ca^2+^ buffers are probably somewhat more effective. The apparent association constant for BAPTA with Ca^2+^, for example, increases by a factor of 1.28 at 37°C, as estimated by the method of Marks and Maxfield (1991). It is also possible that the recording procedure itself changes the structure of the cilium. Learning how well the natural conditions support regenerative Ca^2+^ signals will be challenging. Experimental evidence is sparse. In neuronal models, the concentrations of mobile and immobile buffers are typically estimated to be below 100 µM (Bartol et al., 2015; Ohadi and Rangamani 2019), which in the present study was low enough to allow regenerative signals. The endogenous buffers in those models (e.g. calbindin) have equilibrium association constants with Ca^2+^ that are similar to that of BAPTA or lower (Bartol et al., 2015). In cells of the retinal pigment epithelium, resting intraciliary Ca^2+^ is 580–742 nM (Delling et al., 2013), which may be enough to weakly activate PC2 channels even with strong Ca^2+^ buffering (Kleene and Kleene 2017; Liu et al., 2018). Resting Ca^2+^ is higher in the cilium than in the cytoplasm (107 nM; Delling et al., 2013), suggesting that ciliary channels support some Ca^2+^ entry even at rest. While it seems possible that a small initial stimulus could generate a powerful Ca^2+^ signal in the cilium, evidence for this in intact cells is not yet available. A PC2 agonist increases intraciliary Ca^2+^ in mIMCD-3 cells, but the concentration reached was not determined (Ha et al., 2020).

### Subunit Compositions of the Ca^2+^-Conducting Channels

For convenience, the channels studied here are described as PC2 and TRPV4. However, the subunit compositions of these channels are uncertain. The large-conductance channel, referred to here as PC2, has long been suggested to be a heteromultimer of PC2 and a related protein, polycystin-1 (PC1; reviewed in Douguet et al., 2019), although PC2-dependent channels similar to the native channel persist in cells lacking PC1 (Liu et al., 2018). Circumstantial evidence suggests that the native ciliary PC2 channel may also include TRPM3. The native channel requires expression of both PC2 and TRPM3; in the absence of TRPM3, PC2 traffics to the cilium but functional channels are not detectable (Kleene et al., 2019). Furthermore, the native ciliary channel is activated by pregnenolone sulfate, which also activates TRPM3, and inhibited by isosakuranetin, a specific inhibitor of TRPM3 (Kleene et al., 2019).

The channel referred to here as TRPV4 is activated by GSK1016790A, a TRPV4 agonist (Willette et al., 2008), and inhibited by the selective TRPV4 antagonist HC-067047 (Willette et al., 2008; Everaerts et al., 2010; Siroky et al., 2017). The presence of TRPV4 in renal primary cilia is established (Köttgen et al., 2008; Du et al., 2012; Zhang et al., 2013; Lee et al., 2015; Siroky et al., 2017; Kohli et al., 2017). In the presence of extracellular Ca^2+^, the complex rectification of the current-voltage relation for the ciliary current activated by GSK1016790A (Figure 7C, gray) is the same as that described for TRPV4 (Voets et al., 2002). Exogenously expressed PC2 and TRPV4 can form a heterotetrameric channel with a 2:2 stoichiometry (Stewart et al., 2010). In the plasma membrane of renal epithelial cells, TRPV4 can interact with PC2 (Köttgen et al., 2008; Du et al., 2012; Zhang et al., 2013) and form channels with a conductance of 23 pS (Zhang et al., 2013; Saigusa et al., 2019). In primary cultures from human kidneys, GSK1016790A activates a channel of ∼20 pS at negative potentials (Tomilin et al., 2018). However, channels with a similar conductance were not seen in this study. Instead, the TRPV4 agonist activated channels with conductances of 39 pS at negative potentials and 90 pS at positive potentials (Figure 7C). These are typical values for TRPV4 channels (30–60 pS at negative potentials, 80–100 pS at positive potentials; Nilius et al., 2004; Garcia-Elias et al., 2014). TRPV4 appears to play several roles in renal function (reviewed in Pochynyuk et al., 2013).

### Relationship Between Intraciliary Ca^2+^ and cAMP Signals

It has been suggested that Ca^2+^ fluxes through ciliary ion channels, including PC2, may function to prevent cystogenesis in healthy renal epithelial cells (Jin et al., 2014b; Chebib et al., 2015; Wang et al., 2018). In the cell as a whole, Ca^2+^ and cAMP influence the cellular proliferation and fluid secretion that characterize PKD (reviewed in Calvet 2015). cAMP inhibits cystogenesis in normal cells but promotes it in cystic cells (Hanaoka and Guggino 2000; Yamaguchi et al., 2000). In cultured human renal cells from ADPKD patients, expression of adenylate cyclases AC5 and AC6 is increased, as is the basal cAMP level (Pinto et al., 2012). In mice with PC1 or PC2 knocked out in the kidney, concurrent elimination of AC6 or AC5 (respectively) reduces cystogenesis (Rees et al., 2014; Wang et al., 2018). Cystic cells often have depressed cytoplasmic Ca^2+^ (Yamaguchi et al., 2006; Zaika et al., 2013; Jin et al., 2014b; Tomilin et al., 2018; but see also; Cabrita et al., 2021), and increasing this Ca^2+^ can restore a normal response to cAMP (Yamaguchi et al., 2006; Gradilone et al., 2010; Zaika et al., 2013). Within the cilium, Ca^2+^ may influence the level of cAMP (Wachten and Mick 2021), perhaps by inhibiting AC5 and AC6. (On exogenous expression, each of these shows steadily decreasing activity as internal Ca^2+^ is increased from 0.1 µM to 1 mM (Guillou et al., 1999; Hu et al., 2002).) The primary cilia of renal epithelial cells express AC5 and AC6 (Raychowdhury et al., 2009; Chien et al., 2010; Choi et al., 2011; Mick et al., 2015; Wang et al., 2018), and the cyclases interact in the cilium with PC2 (Choi et al., 2011; Wang et al., 2018). One can thus propose that Ca^2+^ entering through PC2 channels might suppress the cyclases and thus the synthesis of cAMP (Wang et al., 2018). Consistent with this, deletion of PC2 increased forskolin-stimulated intracellular cAMP levels. This was corrected by reexpression of wild-type PC2 but not by a mutant lacking Ca^2+^ channel activity (Choi et al., 2011). Although the relevant proteins form a complex in the cilium, their effects on cystogenesis are not known to reside there. It is known that cAMP in the cilium has functions distinct from those in the cytoplasm (Sherpa et al., 2019; Truong et al., 2021).

### Possible Mechanisms for Initiating a Regenerative Ciliary Ca^2+^ Signal

It also remains to learn what stimuli might initiate an increase in intraciliary Ca^2+^, perhaps regenerative, *in vivo*. Such an increase has been reported on deflection of the cilium (Su et al., 2013; Jin et al., 2014a). However, it is debated whether a ciliary Ca^2+^ increase is mediated by ciliary channels or by backflow of Ca^2+^ from the cell body into the cilium (Delling et al., 2016). In mIMCD-3 cells, a soluble *N*-terminal fragment of PC1 activates PC2 channels and initiates an influx of Ca^2+^ into the primary cilium (Ha et al., 2020). Also in mIMCD-3 cells, pregnenolone sulfate, a natural metabolite, activates PC2 channels but at much higher concentrations than are present *in vivo* (Kleene et al., 2019).

An unexplored possibility is that TRPM3 and TRPV4 may be osmotically activated in the cilium. TRPM3 and TRPV4 are typically activated by hypoosmolality (Oberwinkler and Philipp 2014; Toft-Bertelsen and MacAulay 2021), although this has not been demonstrated in the cilium. TRPM3, TRPV4, and the primary cilium are all required for a normal osmotic response in renal epithelial cells, and that function of TRPM3 appears to reside in the cilium (Siroky et al., 2017). Activation of TRPM3 and TRPV4 could be beneficial in PKD. In a cell line derived from the cortical collecting duct, TRPV4 mediates a Ca^2+^ influx in response to hypotonicity (Wu et al., 2007). Reducing the osmolality of the renal filtrate that bathes the cilia might osmotically activate TRPM3 and TRPV4 in the cilium. This could in turn increase ciliary Ca^2+^, which could then inhibit the ciliary cyclases, reduce ciliary cAMP, and slow the progression of PKD. The increased ciliary Ca^2+^ could secondarily activate PC2, but such a model does not explain why the channel function of PC2 in particular is needed to prevent cystogenesis. In a model of autosomal recessive PKD, increased water intake, which can reduce the osmolality of the urine, did slow the progression of the cystic disease (Nagao et al., 2006). Increased water intake has long been discussed as a potential therapy for PKD (Nagao et al., 2006; Torres et al., 2009; Wang et al., 2013) and has been the basis of multiple clinical trials. If such trials are effective, it will be useful to evaluate the roles, if any, of ciliary osmosensitive TRP channels. To date, there is no evidence that defects in TRPM3 or TRPV4 impact renal function. Defects in TRPV4 do not lead to cyst formation in mouse or zebrafish (Köttgen et al., 2008), and existing studies of mice lacking TRPM3 (Vriens et al., 2011; Hughes et al., 2012; Brown et al., 2015; Vandewauw et al., 2018) have not commented on renal function. It is conceivable that TRPM3 and TRPV4 comprise a redundant, fail-safe system such that elimination of either channel can be compensated by the remaining channel.

## Data Availability

The original contributions presented in the study are included in the article/Supplementary Material, further inquiries can be directed to the corresponding author.
