# Erratum: Regenerative calcium currents in renal primary cilia

**DOI:** 10.3389/fphys.2024.1396473

**Published:** 2024-03-18

**Authors:** Frontiers Production Office

**Affiliations:** Frontiers Media SA, Lausanne, Switzerland

**Keywords:** primary cilium, polycystic kidney disease, polycystin-2, PC2, TRPV4, calcium signaling

Due to a production error, there was a mistake in Figure 10 as published. The label under “TRPV4” is missing three characters reading as “Ca^2+^ N^+^.” The corrected label under “TRPV4” should read as “Ca^2+^/K^+^, Na^+^.” The corrected Figure 10 appears below.

**FIGURE 10:**
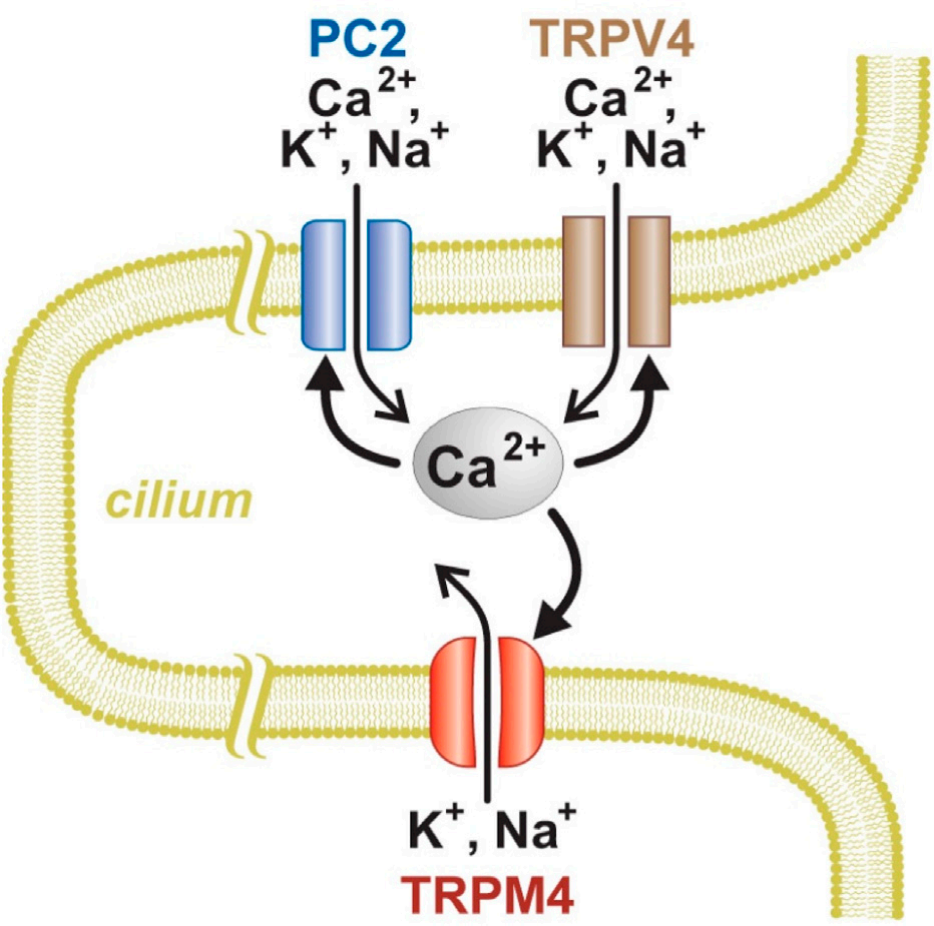
Model of Ca^2+^ signaling and amplification in renal primary cilium. PC2 and TRPV4, but not TRPM4, can conduct Ca^2+^ into the cilium. An increase in intraciliary Ca^2+^ can further activate all three of the channel types.

The publisher apologizes for this mistake.

The original version of this article has been updated.

