# Supplementary material for: Regenerative Calcium Currents in Renal Primary Cilia
Source: Front Physiol. 2022 May 10;13:894518. doi: 10.3389/fphys.2022.894518 (PMC9127361; doi:10.3389/fphys.2022.894518)
Supplement: Supplementary file 1 [file Presentation1.pdf]

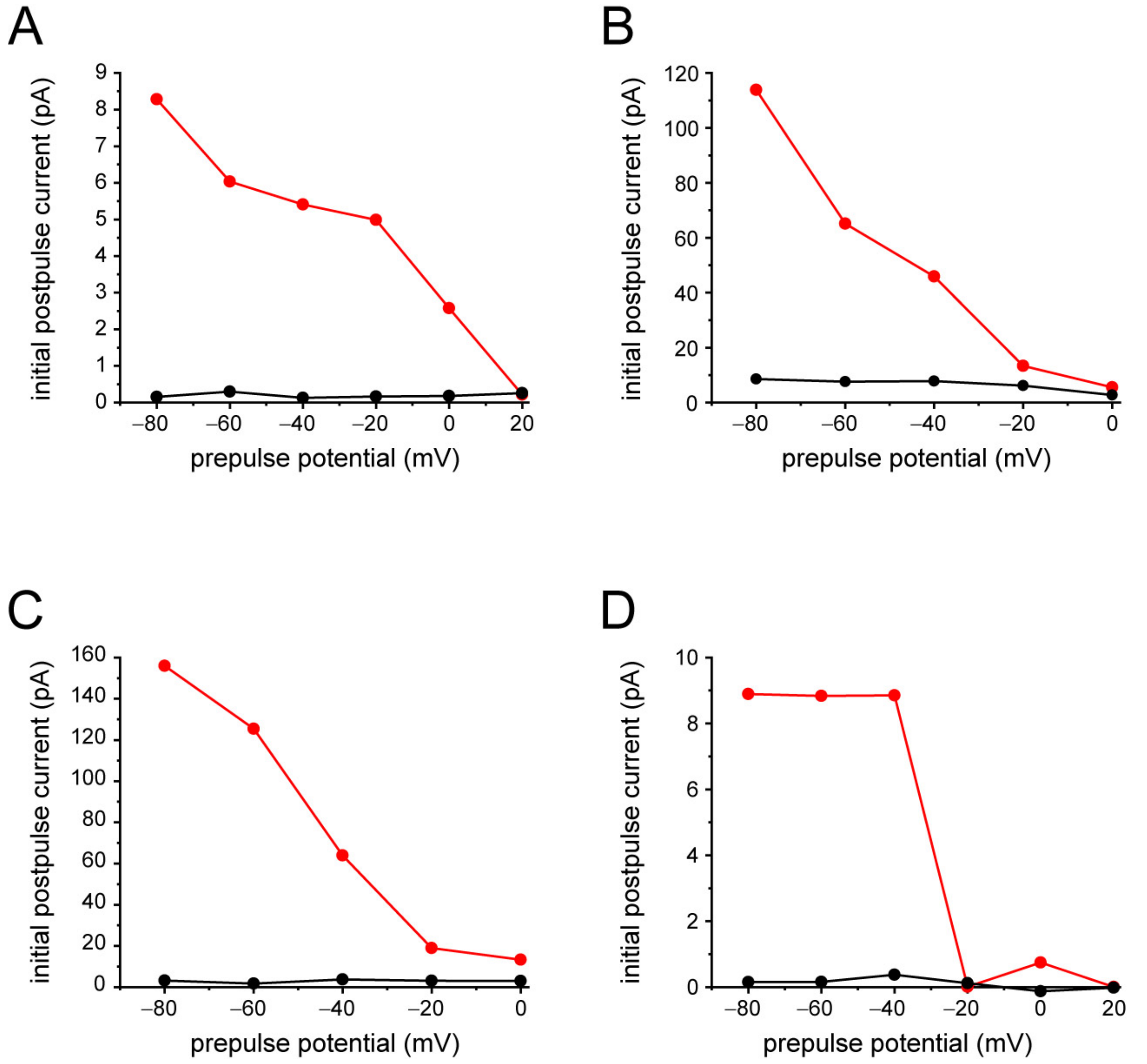

Supplementary Figure S1. Transient PC2 currents are greater with reduced internal Ca^{2+} buffering (0.1 mM BAPTA compared to 2 mM BAPTA). Recordings are from four representative cilia (A-D). For each, the mean current over the first 3 s of a postpulse to +60 mV was measured with 0.1 mM (red) or 2 mM (black) internal BAPTA. No control currents have been subtracted. The *x*-axis shows the values of the voltage prepulses (10 s). In A and D, each cilium had a single active PC2 channel. Four of the original recordings for C are shown in Figure 1A. Four of the original recordings for D are shown in Figure 1B.

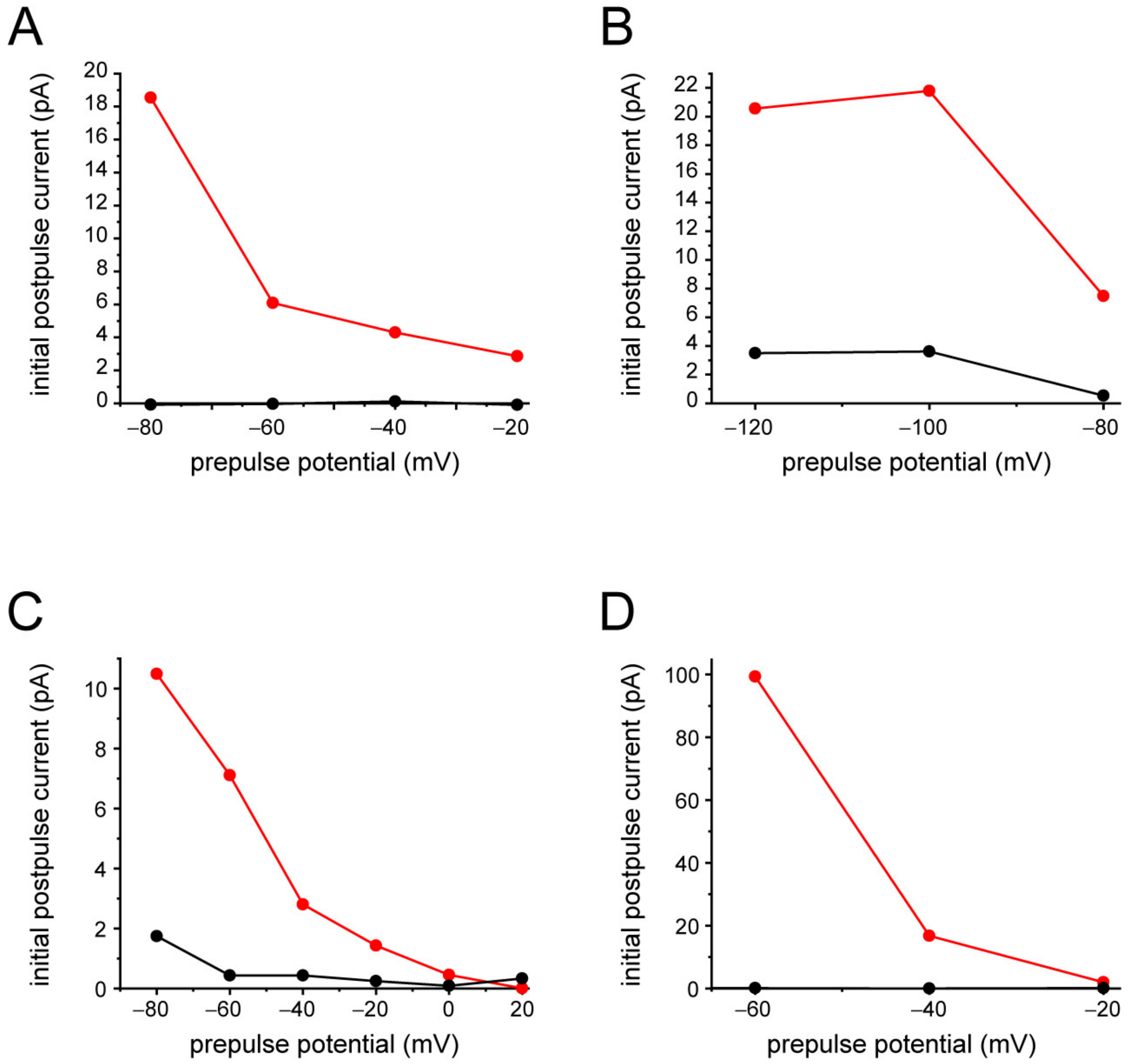

Supplementary Figure S2. Transient PC2 currents are greater with slower Ca^{2+} buffering (0.25 mM EGTA compared to 0.25 mM BAPTA). Recordings are from four representative cilia (A-D). For each, the mean current over the first 3 s of a postpulse was measured with 0.25 mM internal EGTA (red) or 0.25 mM internal BAPTA (black). No control currents have been subtracted. The *x*-axis shows the values of the voltage prepulses (10 s). Postpulse voltages were +60 mV (A), +40 mV (B), +60 mV (C), and +20 mV (D). The original recordings for D are shown in Figure 3A.
